# Prestroke Cognitive Impairment: Frequency and Association With Premorbid Neuropsychiatric, Functional, and Neuroimaging Features

**DOI:** 10.1161/STROKEAHA.123.045344

**Published:** 2024-05-31

**Authors:** Francesco Mele, Ilaria Cova, Alessia Nicotra, Giorgia Maestri, Emilia Salvadori, Valentina Cucumo, Federico Masserini, Martina Martelli, Simone Pomati, Pierluigi Bertora, Leonardo Pantoni

**Affiliations:** Neurology Unit, Luigi Sacco University Hospital, Milan, Italy (F. Mele, I.C., A.N., G.M., V.C., S.P., L.P.).; Department of Clinical and Biomedical Sciences, Neuroscience Research Center, University of Milan, Italy (E.S., F. Masserini, M.M., P.B., L.P.).

**Keywords:** cognitive dysfunction, ischemic stroke, neuroimaging, prevalence, white matter

## Abstract

**BACKGROUND:**

Some patients with stroke have prestroke cognitive impairment (pre-SCI), but its etiology is not clear. The aim of this cross-sectional study was to assess the frequency of pre-SCI and its association with premorbid neuropsychiatric, functional, and neuroimaging features.

**METHODS:**

Patients hospitalized in stroke unit with an informant who could complete IQCODE (Informant Questionnaire for Cognitive Decline in the Elderly) were included. Pre-SCI was diagnosed if the IQCODE score was >3.3. Prestroke assessment also included NPI-Q (Neuropsychiatric Inventory Questionnaire), the basic Activities of Daily Living and Instrumental Activities of Daily Living scales, and the Clinical Dementia Rating scale. A multivariate logistic regression model was used to evaluate the association of pre-SCI with age, sex, education, arterial hypertension, atrial fibrillation, white matter lesions, cerebral microbleeds, and pathological medial temporal lobe atrophy.

**RESULTS:**

IQCODE was available in 474 of 520 patients (91.2%; 45% women; mean age 75.5±13.3 years). Pre-SCI had a prevalence of 32.5% and was associated with prestroke NPI-Q (pre-SCI absent versus present, 1.7±2.3 versus 5.5±4.9; *P*<0.001), Activities of Daily Living scale (0.3±0.8 versus 1.8±1.9; *P*<0.001), Instrumental Activities of Daily Living scale (0.6±1.3 versus 3.8±4.0; *P*<0.001), and Clinical Dementia Rating scale score (0.7±1.7 versus 7.2±6.2; *P*<0.001). In the 271 patients with a magnetic resonance imaging available, the multivariate logistic regression showed that age (odds ratio [OR], 1.05 [95% CI, 1.62–9.73]), white matter lesions (OR, 1.26 [95% CI, 1.003–1.58]), and a pathological medial temporal lobe atrophy score (OR, 3.97 [95% CI, 1.62–9.73]) were independently associated with pre-SCI. In the 218 patients with ischemic stroke, white matter lesions (OR, 1.34 [95% CI, 1.04–1.72]) and medial temporal lobe atrophy (OR, 3.56 [95% CI, 1.38–9.19]), but not age, were associated with pre-SCI.

**CONCLUSIONS:**

One-third of patients admitted to a stroke unit have pre-SCI that is associated with preexisting neuropsychiatric symptoms and functional performance. White matter lesions and medial temporal lobe atrophy are associated with pre-SCI, suggesting that both small vessel disease and neurodegeneration might be involved in its etiology.

After a stroke, many patients develop cognitive deficits that require lifelong assistance. The development and the severity of poststroke cognitive impairment depend not only on the characteristics of the acute lesion, demographic factors, and comorbidities, but also on the prestroke cognitive status. The cognitive impairment that precedes a stroke is referred to as prestroke cognitive impairment (pre-SCI). In a meta-analysis that included 8 hospital-based and 3 population-based studies, the pooled prevalence of prestroke dementia was 14.4% in hospital-based studies and 9.1% in population-based cohorts, with significant interstudy variability.^1^ When including the milder forms of cognitive impairment, prevalence estimates are higher, reaching 24% in some studies.^2,3^ Part of the variability in the epidemiology of pre-SCI depends on the lack of a standardized diagnosis. Some studies have relied on the medical history or clinical interview for the definition of pre-SCI,^4,5^ while others have used informant-based questionnaires,^2,3^ which allow a more reproducible assessment. Among them, the most commonly used tool is the IQCODE (Informant Questionnaire on Cognitive Decline in the Elderly).^6^

Pre-SCI is hypothesized to be consequent to both vascular and degenerative processes, but this is not easy to document premortem. In this regard, cerebrospinal fluid or plasma biomarkers could be of great help, but their use in the acute phase of stroke may be problematic in clinical practice. Neuroimaging may represent a valuable aid on the contrary, since it is routinely performed in this setting and allows the assessment of different markers of chronic vascular changes (such as white matter lesions [WMLs], cerebral microbleeds, and perivascular spaces) and of degenerative processes (medial temporal lobe atrophy [MTLA]). Aim of this prospective study was to assess the frequency of pre-SCI and its relation with premorbid behavioral and functional features and with vascular and degenerative neuroimaging factors.

## METHODS

All patients hospitalized for an acute cerebrovascular event in the stroke unit of the Luigi Sacco Hospital in Milan, Italy, from April 2019 to December 2022 were prospectively screened for inclusion in the study, and those who had an informant available during hospitalization were included. Enrollment was stopped between March and August 2020 and between November 2020 and October 2021 because of the COVID-19 pandemic reorganization of the service. Two trained neuropsychologists administered to informants the 16-item IQCODE^7^ for the assessment of prestroke cognitive status, NPI-Q (Neuropsychiatric Inventory Questionnaire)^8^ for the assessment of prestroke neuropsychiatric symptoms, the basic Activities of Daily Living^9^ and Instrumental Activities of Daily Living^10^ scales for the assessment of prestroke functional abilities, and the 8-item Clinical Dementia Rating (CDR) scale^11^ for a combined assessment of prestroke cognitive, behavioral, and functional status. The IQCODE score^7^ varies from 0 to 5, and pre-SCI was defined by a score >3.3, as suggested by a Cochrane review.^12^ The NPI-Q investigates 12 domains (delusions, hallucinations, agitation/aggression, dysphoria/depression, anxiety, euphoria/elation, apathy/indifference, disinhibition, irritability/lability, aberrant motor nighttime behavior, and appetite/eating), and its severity score varies from 0 to 36.^8^ For Activities of Daily Living and Instrumental Activities of Daily Living scales, the number of items that had been lost by the patient before the index event was registered. The extended version of the CDR scale investigates 8 domains that are related to cognitive, functional, and behavioral features (memory, orientation, judgment and problem solving, community affairs, home and hobbies, personal care, language, and behavior). Each domain has its own score that ranges from 0 to 3. The sum of the scores obtained in the 8 domains is called sum of boxes and ranges from 0 to 24.^11^ Demographic and clinical variables, including age, sex, education, vascular risk factors, and the type and etiology of the acute cerebrovascular event, were registered. Patients underwent brain computed tomography (CT) or magnetic resonance imaging (MRI) as needed in clinical practice. CT imaging was available for all patients, while MRI (1.5T magnet) was available for 271 patients (57%). The evaluation of neuroimaging features was done by 1 trained certified neurologist who was blind to the assessment of preevent clinical status. Table 1 details the scales used for the assessment of the neuroimaging features.

**Table 1.**
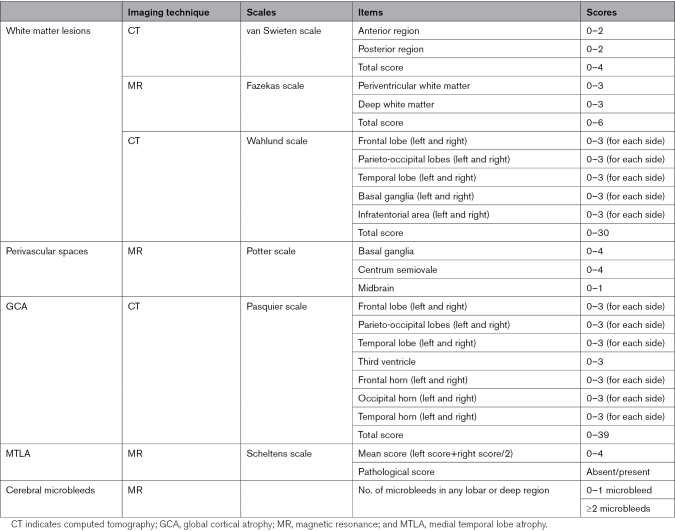
Neuroimaging Assessment

All patients signed an informed consent to undergo any assessment needed in clinical practice, including neuroimaging. The local institutional review board subsequently granted approval for the retrospective analysis of the data. All the procedures were performed in accordance with the Declaration of Helsinki. The corresponding author had full access to all the data in the study and took responsibility for its integrity and the data analysis. The data that support the findings of this study are available from the corresponding author upon reasonable request. The article follows the Strengthening the Reporting of Observational Studies in Epidemiology guidelines.^13^

### Statistical Analysis

The scores obtained on each item of the van Swieten,^14^ Fazekas,^15^ and Potter^16^ scales were used as ordinal variables. The total scores of the van Swieten, Fazekas, Wahlund,^17^ and Pasquier^18^ scales and the mean score of the Scheltens scale^19^ were used as continuous variables. After adjustment for age, sex, and education (as suggested by Velickaite et al),^20^ the presence of a pathological MTLA score was used as a categorical binary variable. The number of cerebral microbleeds was categorized into 2 groups (<2 versus ≥2 microbleeds) and used as a categorical variable. All continuous variables were tested for normality using the Kolmogorov-Smirnov test.

In the whole cohort of patients, a comparison of demographic, clinical, and CT-based neuroimaging (van Swieten, Wahlund, and Pasquier scales) variables was made between the group of patients without pre-SCI and the group of patients with pre-SCI, using χ^2^ test for categorical variables and Mann-Whitney *U* test for ordinal and non-normally distributed continuous variables.

In the subgroup of patients who had an MRI available, binomial logistic regression models were created for each neuroimaging variable, together with age, sex, education, hypertension, and atrial fibrillation as covariates and pre-SCI as the dependent variable. In these models, ordinal imaging variables were computed according to the reverse Helmert coding, in which every level of a variable is compared with the mean of the previous levels.

In the subgroup of patients with an MRI available, a second step consisted in the creation of a global logistic regression model, with age, sex, education, hypertension, atrial fibrillation, 1 measure of WML (the scale, among the 3 used, that had shown the strongest association with pre-SCI across the previous models), cerebral microbleeds, a measure of neurodegeneration (the MTLA pathological score), and a measure of global cortical atrophy (Pasquier scale) as covariates and pre-SCI as the dependent variable. Since global cortical atrophy cannot be clearly attributed to either a degenerative or a vascular mechanism, in a successive binomial logistic regression model, the Pasquier scale was excluded from the analysis, while retaining all the other variables. This last logistic regression model was first applied to the whole cohort of patients with MRI and then, separately, to the ischemic and the hemorrhagic stroke subgroups, under the hypothesis that different mechanisms may underlie pre-SCI in these subgroups. All statistical analyses were performed with the IBM SPSS software (version 28), using a *P* value threshold of 0.05 to ascertain statistical significance.

## RESULTS

### Demographic and Clinical Features and Frequency of Pre-SCI

Five hundred twenty consecutive patients were screened during the study period. For 46 patients (8.8%), IQCODE could not be administered during the hospitalization because no informant was available. The remaining 474 patients (91.2%) were included in the study. The reason for hospitalization was an ischemic stroke for 386 patients (81%), a hemorrhagic stroke for 52 patients (11%), and a transient ischemic attack for 36 patients (8%). Ischemic stroke etiologies were large artery atherosclerosis in 76 patients (20%), small vessel disease in 39 patients (10%), cardioembolism in 112 patients (29%), other definite causes in 15 patients (4%), cryptogenic in 102 patients (27%), multiple possible causes in 27 patients (7%), and undetermined due to insufficient assessment in 15 patients (4%). Of the 474 patients included, 212 (45%) were women. Mean age was 75.5 years (SD, 13.3), and mean education was 9.7 years (SD, 4.8). Mean National Institutes of Health Stroke Scale score on admission was 5.9 (SD, 6.3). Hypertension was present in 345 patients (73%), atrial fibrillation in 100 patients (21%), diabetes in 128 patients (27%), dyslipidemia in 280 patients (59%), and a current or previous history of smoking in 223 patients (49%). Based on IQCODE (cutoff score, 3.3), pre-SCI was present in 154 patients (32.5%). No statistically significant difference in the prevalence of pre-SCI was seen across the 3 cerebrovascular event categories (33.7% in patients with ischemic stroke, 30.8% in patients with hemorrhagic stroke, and 22.2% in patients with transient ischemic attack) or across the 3 main classes of ischemic stroke etiology (38.5% in large artery atherosclerosis, 30.8% in small vessel disease, and 36.8% in cardioembolism).

### Demographic, Clinical, and CT-Based Neuroimaging Features Associated With Pre-SCI

Table 2 shows the demographic, clinical, and neuroimaging features in the whole cohort of patients and the comparison between the group of patients with pre-SCI and the group of patients without pre-SCI. Patients with pre-SCI were older and more often women and had lower education and a higher prevalence of hypertension and atrial fibrillation, compared with patients without pre-SCI. All CT-based neuroimaging variables (van Swieten, Wahlund, and Pasquier scale scores) showed a different distribution between patients with pre-SCI and patients without pre-SCI, being more severe in the pre-SCI group. As shown in the Figure, compared with patients without pre-SCI, patients with pre-SCI had higher scores on NPI-Q, had lost independence in more Activities of Daily Living and Instrumental Activities of Daily Living scales, and had higher scores on Clinical Dementia Rating scale–sum of boxes. The scores obtained in each of the CDR domains were also higher in patients with pre-SCI, compared with patients without pre-SCI, as shown in Table S1.

**Table 2.**
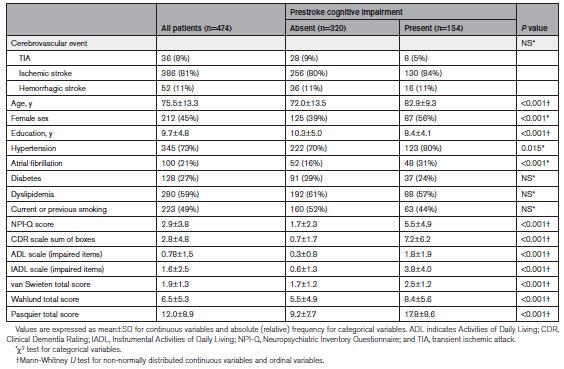
Demographic and Clinical Features in the Whole Cohort of Patients and in Patients With or Without Prestroke Cognitive Impairment

**Figure.**
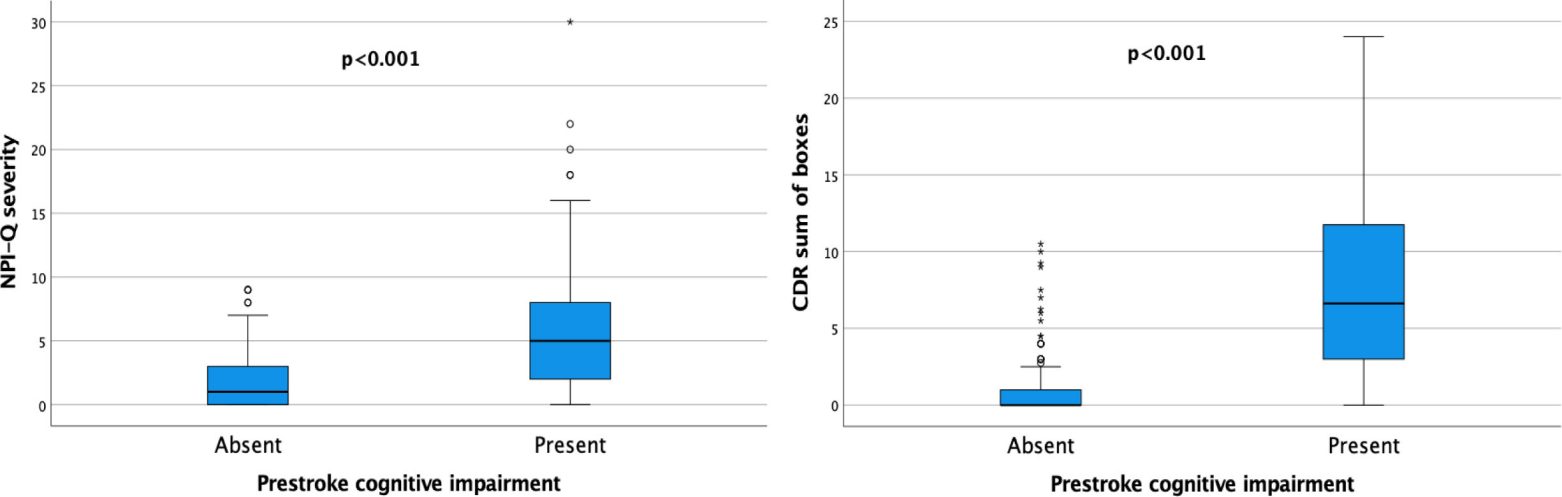
**Neuropsychiatric symptoms and cognitive and functional impairment in daily living in patients with and without prestroke cognitive impairment.** CDR indicates Clinical Dementia Rating; and NPI-Q, Neuropsychiatric Inventory Questionnaire.

### Neuroimaging Features Associated With Pre-SCI in Patients With an MRI Available

As shown in Table 3, patients who underwent an MRI (n=271) were more often men, were younger, had a higher education, and were less likely to be affected by atrial fibrillation, compared with patients who did not have an MRI study (n=203). Among the 271 patients who had an MRI, 71 patients had a pre-SCI (26.2%).

**Table 3.**
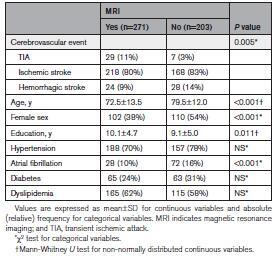
Comparison of Demographic and Clinical Features Between Patients With an MRI and Patients Without an MRI Available

Table 4 shows the distributions of imaging scores in patients with and without pre-SCI, in the group of patients who had an MRI available. On regression analyses, pre-SCI was significantly associated with the anterior (odds ratio [OR], 2.36 for score 2 [95% CI, 1.11–5.02]) van Swieten score; with the periventricular (OR, 2.21 for score 2 [95% CI, 1.01–4.83]; 3.14 for score 3 [95% CI, 1.33–7.42]), deep (OR, 3.09 for score 3 [95% CI, 1.14–8.42]), and total (OR, 1.35 for each 1-point increase [95% CI, 1.11–1.64]) Fazekas scores; with the total Wahlund score (OR, 1.08 for each 1-point increase [95% CI, 1.02–1.14]); with the total Pasquier score (OR, 1.09 for each 1-point increase [95% CI, 1.05–1.14]); with the mean Scheltens score (OR, 1.89 for each 1-point increase [95% CI, 1.38–2.59]); with a pathological MTLA (OR, 5.25 [95% CI, 2.25–12.25]); and with the presence of at least 2 cerebral microbleeds (OR, 2.42 [95% CI, 1.14–5.15]).

**Table 4.**
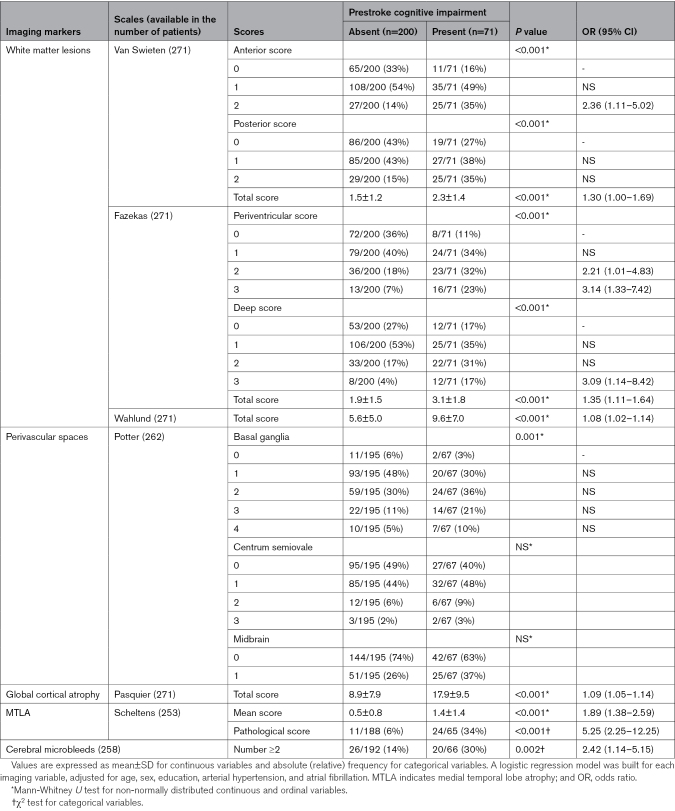
Imaging Parameters in the Group of Patients With MRI (n=271), Categorized According to the Presence or Absence of Prestroke Cognitive Impairment

Table 5 shows the results of the final regression models. When all neuroimaging variables were included (model 1), the Pasquier total score was the only factor associated with pre-SCI (OR, 1.07 for each 1-point increase [95% CI, 1.001–1.14]). When Pasquier total score was excluded from the model (model 2), WML (OR, 1.26 for each 1-point increase in the Fazekas total score [95% CI, 1.003–1.58]), MTLA (OR, 3.97 for a pathological score [95% CI, 1.62–9.73]), and age (OR, 1.05 for each 1-year increase [96% CI, 1.01–1.09]) were associated with pre-SCI. In the subgroup of 218 patients with ischemic stroke, the Fazekas total score (OR, 1.34 for each 1-point increase [95% CI, 1.04–1.72]) and MTLA (OR, 3.56 for a pathological Scheltens score [95% CI, 1.38–9.19]), but not age (OR, 1.037 [95% CI, 0.996–1.079]), were associated with pre-SCI, whereas in the hemorrhagic stroke and transient ischemic attack subgroups, no statistically significant associations were found, as shown in Table S2.

**Table 5.**
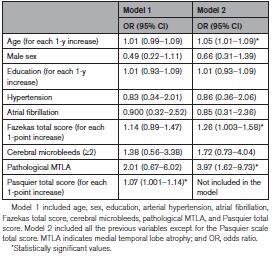
Logistic Regression Models of Demographic, Clinical, and Neuroimaging Variables Associated With Prestroke Cognitive Impairment

## DISCUSSION

Our study shows that one-third of patients admitted to a stroke unit are affected by pre-SCI. Previous studies have indicated lower prevalence estimates of pre-SCI, but some of these have used 4.0 as the IQCODE cutoff for the definition of pre-SCI,^21–23^ thus restricting the investigation to the most severe forms of pre-SCI. When comparing our study with others that used the same IQCODE cutoff,^2,3^ our study still has a higher prevalence of pre-SCI, which could be explained by the higher age of our cohort, but also by some differences in the inclusion criteria. Mok et al^2^ have studied a small number of patients with an ischemic stroke associated with small vessel disease, excluding patients with other stroke etiologies. Caratozzolo et al have studied a cohort of patients that shares many similarities with our cohort, being based in the same geographic area. However, they have excluded all patients with a previous diagnosis of dementia (based on the Diagnostic and Statistical Manual of Mental Disorders, Third Edition).^3,24^ This approach, focusing on the milder or undiagnosed forms of pre-SCI, lowers the prevalence estimates of pre-SCI. Our study also found an association of pre-SCI with measures of prestroke neuropsychiatric symptoms and functional impairment. These data strengthen the role of IQCODE in correctly identifying patients with pre-SCI. Previous studies had already shown the association of an IQCODE-based definition of pre-SCI with NPI-Q measures, loss of function on Activities of Daily Living and Instrumental Activities of Daily Living scales, and modified Rankin Scale,^3,21,25^ but the association with the CDR domains and total score had never been studied before and underlines how pre-SCI involves different cognitive, behavioral, and functional aspects of prestroke life.

The main focus of our work was the relationship of pre-SCI with neuroimaging markers of vascular disease and neurodegeneration. WMLs are a marker of small vessel disease and have been assessed by means of different visual rating scales over the years. Our results showed that WMLs are associated with pre-SCI independently of age, sex, education, hypertension, and atrial fibrillation and that each of the scales we assessed (van Swieten, Fazekas, and Wahlund scales) may be used in this regard, in line with the already shown good correlation among them.^26^

Two other markers of small vessel disease were included in our assessment, that is, perivascular spaces and microbleeds. Both have been previously investigated, only in patients with atrial fibrillation–related strokes, by Banerjee et al,^27^ who did not find any significant association with pre-SCI. Our data confirm that perivascular spaces are not independently associated with pre-SCI. In our cohort (which included not only cardioembolic strokes but also other etiologies), the presence of at least 2 microbleeds was associated with pre-SCI. This association, however, lost its significance when WMLs were added to the model, suggesting that the latter is a stronger small vessel disease–related predictor of pre-SCI.

Also measures of cerebral atrophy have been previously investigated in relation to pre-SCI,^4,5,27^ and our data confirm the association of both GCA and MTLA with pre-SCI. However, GCA cannot be ascribed uniquely to a vascular or degenerative process as it is a feature of both.^28,29^ In this regard, it is interesting to note that, when we kept GCA in the statistical model, the weight of the other variables disappeared, while it emerged when we excluded GCA. Our interpretation is that GCA is a potent determinant of pre-SCI and of any dementia-related process. However, in the attempt to disentangle the respective role of vascular and degenerative changes in pre-SCI (and in stroke-related cognitive decline in general), GCA should not be taken into account. Noteworthy, when including in the same model measures of WML and MTLA, the association with pre-SCI was significant for both. To our knowledge, this is the first work showing that both WML and MTLA, independently of one another, are associated with pre-SCI and further supports the hypothesis that both small vessel disease and neurodegeneration are involved in its etiology.

Our study has limitations. First, this was a single-center study that needs to be reproduced in multicentric cohorts. Indeed, despite including a relatively large number of patients, hemorrhagic strokes and transient ischemic attack were underrepresented, as expected. The absence of any significant association of pre-SCI with demographic, clinical, and neuroimaging variables in these patients’ groups may, therefore, be due to the low numbers. Similarly, when ischemic strokes were categorized according to the different etiologies, the numbers were too low to show any differences. Second, the use of data derived from routine clinical practice did not allow the use of computerized assessment methods. Another limitation is that not all patients underwent an MRI, introducing a possible selection bias and limiting the assessment of some lesion types and the power and generalizability of the study. Lastly and most relevant, we could not include in our assessment other biomarkers of small vessel disease or neurodegeneration (such as those derived from the blood and cerebrospinal fluid) that, combined with neuroimaging measures, may allow a more precise profiling of pre-SCI.

In conclusion, our study showed that one-third of patients admitted to a stroke unit had pre-SCI, when assessed with a standardized tool, and that pre-SCI is associated with preexisting neuropsychiatric symptoms and functional performance in daily living. We also showed that pre-SCI is associated with neuroimaging biomarkers of both small vessel disease and neurodegeneration, suggesting that both processes are involved in its etiology.

## ARTICLE INFORMATION

### Sources of Funding

None.

### Disclosures

A. Nicotra and G. Maestri have been partially supported by a liberal donation from PIAM Pharmaceutics Italy to the Department of Biomedical and Clinical Sciences, Neuroscience Research Center, University of Milan. The other authors report no conflicts.

### Supplemental Material

Tables S1–S2

## Supplementary Material


