# Supplementary material for: Prestroke Cognitive Impairment: Frequency and Association With Premorbid Neuropsychiatric, Functional, and Neuroimaging Features
Source: Stroke. 2024 May 31;55(7):1869–76. doi: 10.1161/STROKEAHA.123.045344 (PMC11198949; doi:10.1161/STROKEAHA.123.045344)
Supplement: Supplementary file 1 [file str-55-1869-s001.pdf]

Mele et al. Prestroke cognitive impairment: frequency and association with premorbid neuropsychiatric, functional, and neuroimaging features

SUPPLEMENTAL MATERIAL

Table S1. Association of scores on the CDR domains and prestroke cognitive impairment.

CDR domains	Prestroke cognitive impairment		p-value*
	Absent	Present	
Memory	0.07 ± 0.19	0.95 ± 0.77	**<0.001**
Orientation	0.01 ± 0.07	0.70 ± 0.82	**<0.001**
Judgement and problem solving	0.03 ± 0.15	0.74 ± 0.86	**<0.001**
Community affairs	0.20 ± 0.47	1.20 ± 0.95	**<0.001**
Home and hobbies	0.17 ± 0.44	1.24 ± 1.00	**<0.001**
Personal care	0.12 ± 0.47	1.00 ± 1.08	**<0.001**
Behavior	0.07 ± 0.25	0.54 ± 0.77	**<0.001**
Language	0.04 ± 0.15	0.41 ± 0.65	**<0.001**

Values are expressed as mean ± SD
*Mann-Whitney U test

Table S2. Logistic regression of demographic, clinical, and neuroimaging variables associated with prestroke cognitive impairment in patients with different cerebrovascular events.

	**Ischemic stroke**	**Hemorrhagic stroke**	**TIA**
	**OR (95%C.I.)**	**OR (95%C.I.)**	**OR (95%C.I.)**
Age<br>(for each 1-year increase)	1.04 (0.99-1.08)	1.07 (0.85-1.36)	2.17 (0.75-6.28)
Male sex	0.79 (0.34-1.80)	0.01 (0.00-13.26)	52.7 (0.01-474619.24)
Education<br>(for each 1-year increase)	1.02 (0.94-1.10)	0.50 (0.17-1.47)	0.58 (0.13-2.63)
Hypertension	0.75 (0.30-1.88)	0.01 (0.00-28.78)	7207.30 (0.00-5698e)
Atrial fibrillation	1.03 (0.33-3.20)	31.85 (0.06-16058.88)	0.00 (0.00-2.652e)
Fazekas total score<br>(for each 1-point increase)	**1.34 (1.04-1.72)**	0.46 (0.07-3.21)	0.82 (0.13-5.29)
Cerebral microbleeds (≥2)	1.62 (0.59-4.48)	95.21 (0.04-215823.53)	8.25 (0.00-219270245)
Pathological MTLA	**3.56 (1.38-9.19)**	*n.a.*	3706.99(0.00-3924e)

CI, confidence interval; MTLA, medial temporal lobe atrophy; n.a., not applicable; OR, odds ratio; TIA, transient ischemic attack
